# Primary tracheal lymphoepithelioma-like carcinoma in a young non-smoking male: a case report highlighting the diagnostic value of flow-volume loop

**DOI:** 10.3389/fmed.2026.1965664

**Published:** 2026-10-16

**Authors:** Shan Xiao, Sida Chen, Jintao Ou, Yan Shen

**Affiliations:** Department of Pulmonary and Critical Care Medicine, Shenzhen Longgang District Central Hospital, Shenzhen, China

**Keywords:** airway obstruction, case report, flow-volume loop, lymphoepithelioma-like carcinoma, tracheal neoplasms

## Abstract

**Background:**

Primary tracheal lymphoepithelioma-like carcinoma (LELC) is an extremely rare malignancy (<2% of tracheal tumors) strongly associated with Epstein–Barr virus. Tracheal tumors can mimic asthma due to dynamic airway obstruction, often leading to diagnostic delay. The flow-volume loop provides critical clues to central airway obstruction but is frequently overlooked.

**Case Presentation:**

We report a 30-year-old non-smoking male initially misdiagnosed with asthma, whose refractoriness to inhaled corticosteroids prompted spirometry. The flow-volume loop revealed a plateau pattern in both inspiratory and expiratory limbs, consistent with **fixed upper airway obstruction** which guided chest computed tomography (CT) and bronchoscopy to identify a tracheal polypoid mass. Pathological examination confirmed LELC with positive EBV-encoded small RNAs (EBER) *in situ* hybridization. The patient underwent endoscopic resection followed by video-assisted thoracoscopic surgery (VATS) tracheal resection-anastomosis with lymphadenectomy. Postoperative pulmonary function testing at 1-year follow-up demonstrated complete normalization of the flow-volume loop morphology, with FEV_1_ (Forced expiratory volume in 1 s) improved from 0.91L to 3.62L(+298%) and PEF (peak expiratory flow) from 2.03L/s to 6.76 L/s (+233%). The patient remained asymptomatic without recurrence at 1-year follow-up.

**Conclusion:**

This case underscores that, in young patients with asthma-like symptoms refractory to conventional therapy, flow-volume loop analysis should not be deferred, as it can unmask central airway obstruction and expedite the diagnosis of rare tracheal malignancies.

## Introduction

Primary tracheal malignancies account for less than 0.1% of all cancers, of which approximately two-thirds are squamous cell carcinoma or adenoid cystic carcinoma. Lymphoepithelioma-like carcinoma (LELC) is an extremely rare subtype characterized by undifferentiated epithelial tumor cells surrounded by dense lymphocytic infiltration and strong association with Epstein–Barr virus (EBV) infection, particularly prevalent in Asian populations (1–4). Primary tracheal LELC has been reported only in isolated case reports worldwide (4, 5). Due to dynamic airway obstruction, tracheal tumors frequently mimic asthma, leading to diagnostic delay and unnecessary antiasthma therapy. We report a case of primary tracheal LELC in a young non-smoking male, emphasizing the pivotal role of flow-volume loop analysis in unmasking central airway obstruction when asthma therapy fails.

## Case presentation

A 30-year-old male never-smoker, working as a technical engineer without occupational dust or biomass fuel exposure, presented with a 6-month history of recurrent cough and progressive dyspnea, which had acutely worsened over the past day. Dyspnea was prominent at night and during exertion, accompanied by scanty whitish-yellow sputum. Intermittent wheezing was audible, but the patient denied fever, chest pain, hemoptysis, night sweats, or paroxysmal nocturnal dyspnea. His medical history was unremarkable, with no history of atopy, tuberculosis, connective tissue disease, or immunocompromised status. He had no recent travel history. He initially consulted a primary care physician and was empirically diagnosed with asthma, prescribed inhaled budesonide/formoterol (160/4.5 ug, twice daily). After two months of adherence to this regimen, his symptoms showed no improvement.

On physical examination, vital signs were stable (respiratory rate 18/min, oxygen saturation 97% on room air); inspiratory stridor was audible over the trachea, without cyanosis or intercostal retractions. Laboratory findings (all within normal limits unless specified): complete blood count showed WBC 5.48 × 10⁹/L, neutrophils 52.4%, lymphocytes 38.3%, eosinophils 1.5% (absolute count 0.08 × 10⁹/L), hemoglobin 153 g/L, platelets 194 × 10⁹/L. C-reactive protein and erythrocyte sedimentation rate were within normal limits. Epstein–Barr virus serology revealed EBVCA-IgG 34 U/mL (positive), EBNA-IgG >600.0 U/mL (positive), EBVCA-IgM 0.78 U/mL (negative), EB-VCA-IgA 0.27 S/CO (negative), EBNA-IgA 0.54 S/CO (negative), EBEA-IgM 0.06 S/CO (negative). Plasma EBV DNA was detectable (positive). Fractional exhaled nitric oxide (FeNO) was 60 ppb (elevated).

Spirometry with flow-volume loop analysis (Figure 1) demonstrated a plateau pattern in both inspiratory and expiratory limbs, with markedly reduced peak expiratory flow (PEF 2.03 L/s) and peak inspiratory flow (PIF approximately 2.30 L/s), consistent with **fixed upper airway obstruction**. Bronchodilator reversibility testing was positive: pre-bronchodilator FEV₁ was 0.91 L (22.1% predicted), post-bronchodilator FEV₁ was 1.75 L (42.6% predicted), with an absolute increase of 0.84 L (92% increase); however, the plateau pattern persisted on the post-bronchodilator loop. Chest CT revealed a polypoid mass protruding into the tracheal lumen from the anterior wall of the cervical trachea at the third-fourth cervical vertebra (C3-C4) level (Figures 2A,B). Bronchoscopy identified a central polypoid mass, causing near-complete (approximately 95%) luminal occlusion of the cervical trachea (Figure 2C), which was electrosnared endoscopically.

**Figure 1:**
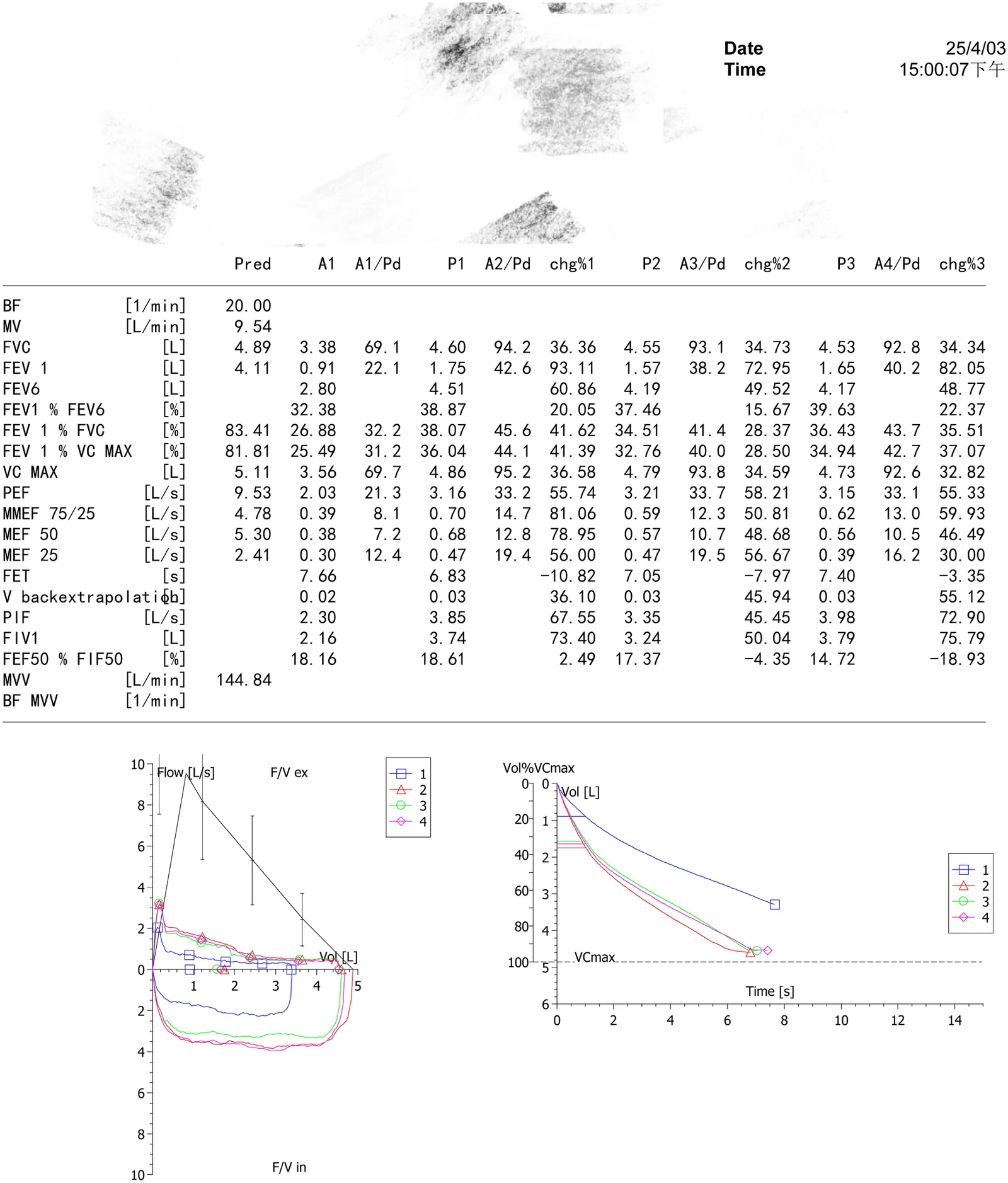
Spirometry flow-volume loop showing a plateau pattern in both inspiratory and expiratory limbs, consistent with fixed upper airway obstruction. The dashed line shows the predicted values.

**Figure 2:**
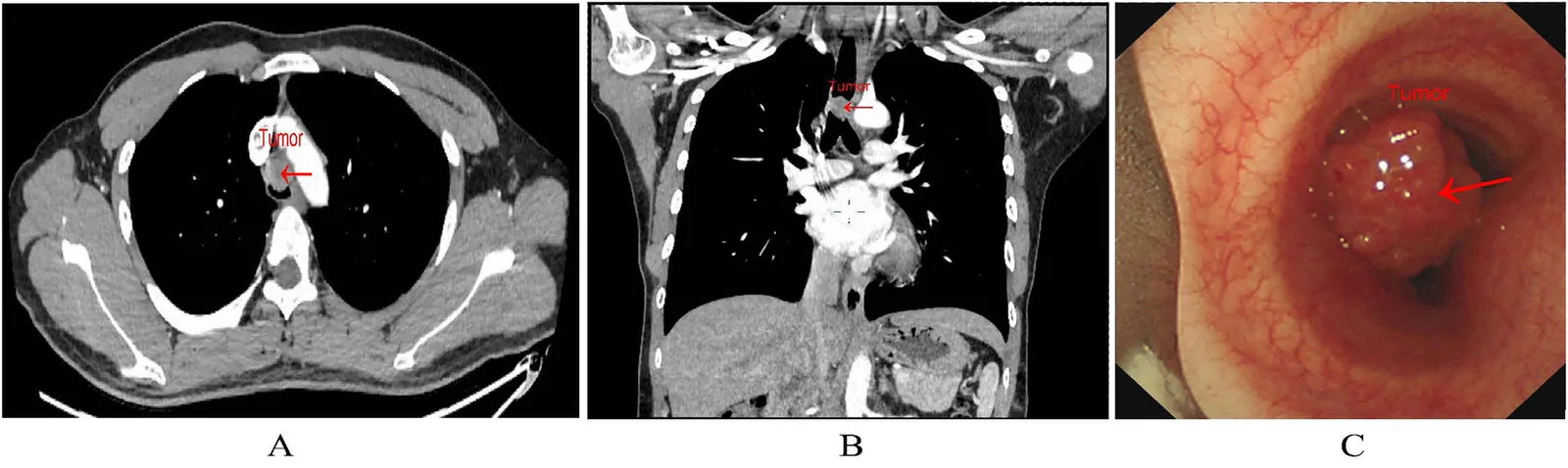
**(A)** axial and **(B)** coronal views of chest computed tomography showing a polypoid mass protruding into the lumen from the anterior wall of the cervical trachea at the C3–C4 level. **(C)** Bronchoscopic image showing a **central** polypoid mass causing near-complete occlusion of the **cervical trachea**.

Histopathological examination showed nests and sheets of malignant cells surrounded by dense lymphocytic infiltration and hematoxylin and eosin staining (Figure 3A). Immunohistochemistry demonstrated diffuse cytoplasmic positivity for CK5/6 and P40 (Figures 3B–D), and negativity for Napsin A, TTF-1, Syn and CgA. The Ki-67 proliferative index was 70%. *In situ* hybridization for EBV-encoded small RNAs (EBER) revealed strong nuclear positivity (Figure 3C). This immunoprofile (CK5/6+, P40+, Napsin A-, TTF-1-, Syn-, CgA-, EBER+) confirms the diagnosis of primary tracheal LELC.

**Figure 3:**
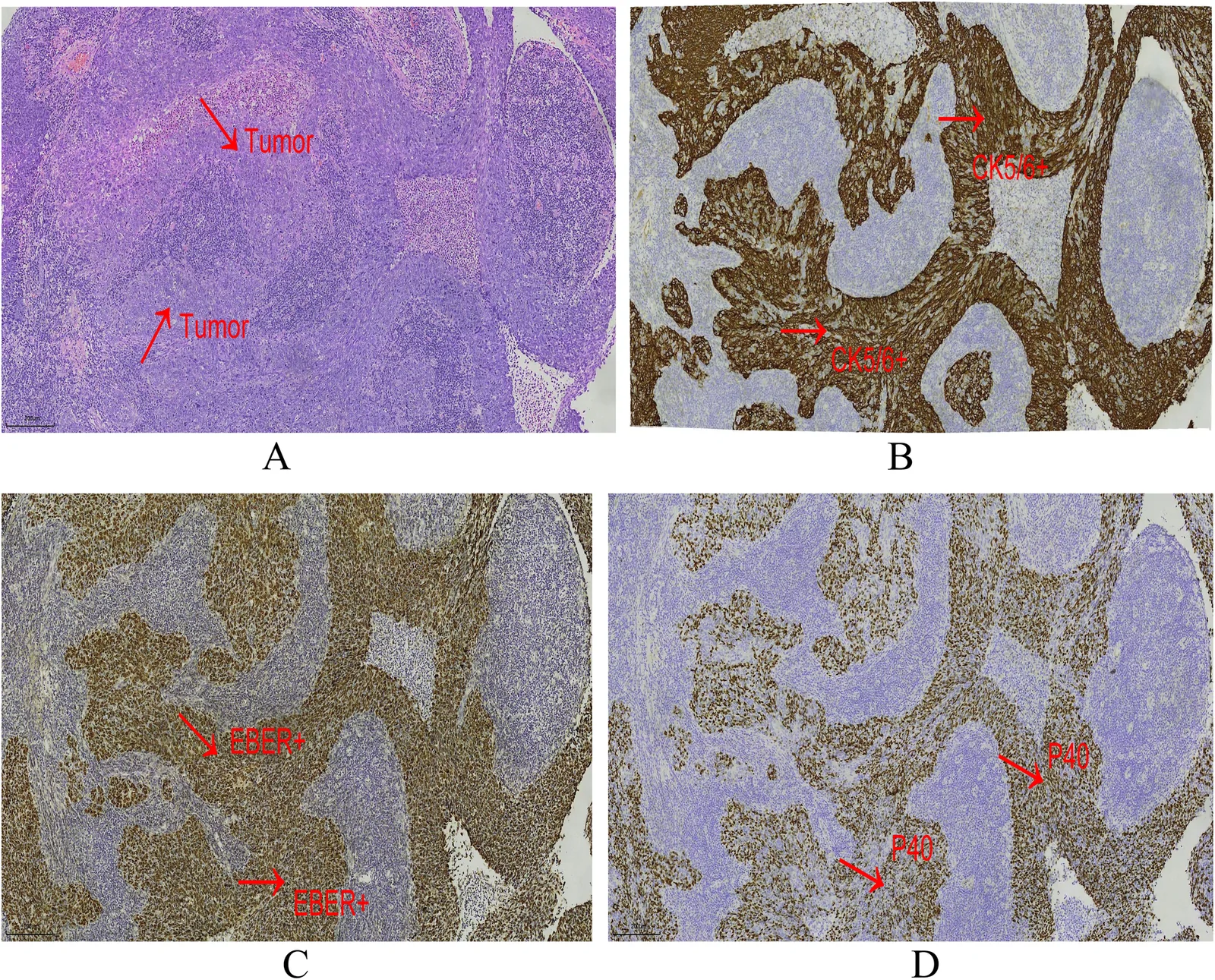
**(A)** Hematoxylin and eosin stain showing that the tumor histology was composed of nests and sheets of malignant cells surrounded by lymphocytic infiltrates. **(B)** Immunohistochemical findings revealing that the tumor cells were immunoreactive to CK5/6. **(C)** The tumor cells showing strong nuclear positivity for EBERs by *in situ* hybridization, suggesting the diagnosis of primary tracheal lymphoepithelioma-like carcinoma. **(D)** Immunohistochemical findings revealing that the tumor cells were immunoreactive to P40.

To exclude a nasopharyngeal primary, the patient underwent fiberoptic laryngoscopy and nasoendoscopy, which demonstrated no abnormal findings in the nasopharynx or upper aerodigestive tract, and 18F-FDG PET/CT, which demonstrated no hypermetabolic lesion in the nasopharynx or elsewhere outside the trachea and regional mediastinal lymph nodes. Based on the solitary tracheal mass, absence of a primary tumor elsewhere, and characteristic immunohistochemical and EBER-positive profile, a diagnosis of primary tracheal LELC was established. We acknowledge that dedicated nasopharyngeal biopsy was not performed, as no suspicious lesion was identified, which represents a diagnostic limitation.

Subsequent 18F-FDG PET/CT detected residual mediastinal lymph node metastasis, prompting salvage VATS tracheal resection-anastomosis with systematic lymphadenectomy. Pathological examination confirmed negative resection margins (R0), with the closest margin approximately 0.2 cm from the tumor. A total of 11 mediastinal lymph nodes were resected, of which 2 were positive for metastatic carcinoma: 1 in station 2R (2R-1LN, 1/1) and 1 in station 4R (4R-5LN, 1/1). The primary tumor measured approximately **2 cm** **×** **1.2 cm** **×** **0.5 cm** and demonstrated **invasion into the cricoid cartilage** without extension beyond the adventitia. No vascular or perineural invasion was identified. Given the absence of a validated TNM staging system specifically established for primary tracheal LELC, the tumor was classified as pT2N2M0/Stage IIIA **by reference to** existing staging systems. We acknowledge the absence of a dedicated tracheal staging system as a limitation. The patient had an uneventful postoperative recovery and received no adjuvant systemic therapy. At 1-year follow-up, the patient remained asymptomatic without evidence of recurrence on imaging. Postoperative pulmonary function testing at 1 year (Figure 4) demonstrated complete normalization of the flow-volume loop morphology, with FEV_1_(Forced expiratory volume in 1 s) improved to 3.62L(89.7% predicted), FVC (Forced vital capacity) to 4.31 L (89.6% predicted), FEV_1_/FVC to 84.1%, and PEF (peak expiratory flow) to 6.76 L/s (71.8% predicted). The plateau pattern had completely resolved, confirming restoration of normal airway dynamics. Plasma EBV DNA remained detectable at low levels (<500 copies/mL), warranting continued surveillance.

**Figure 4:**
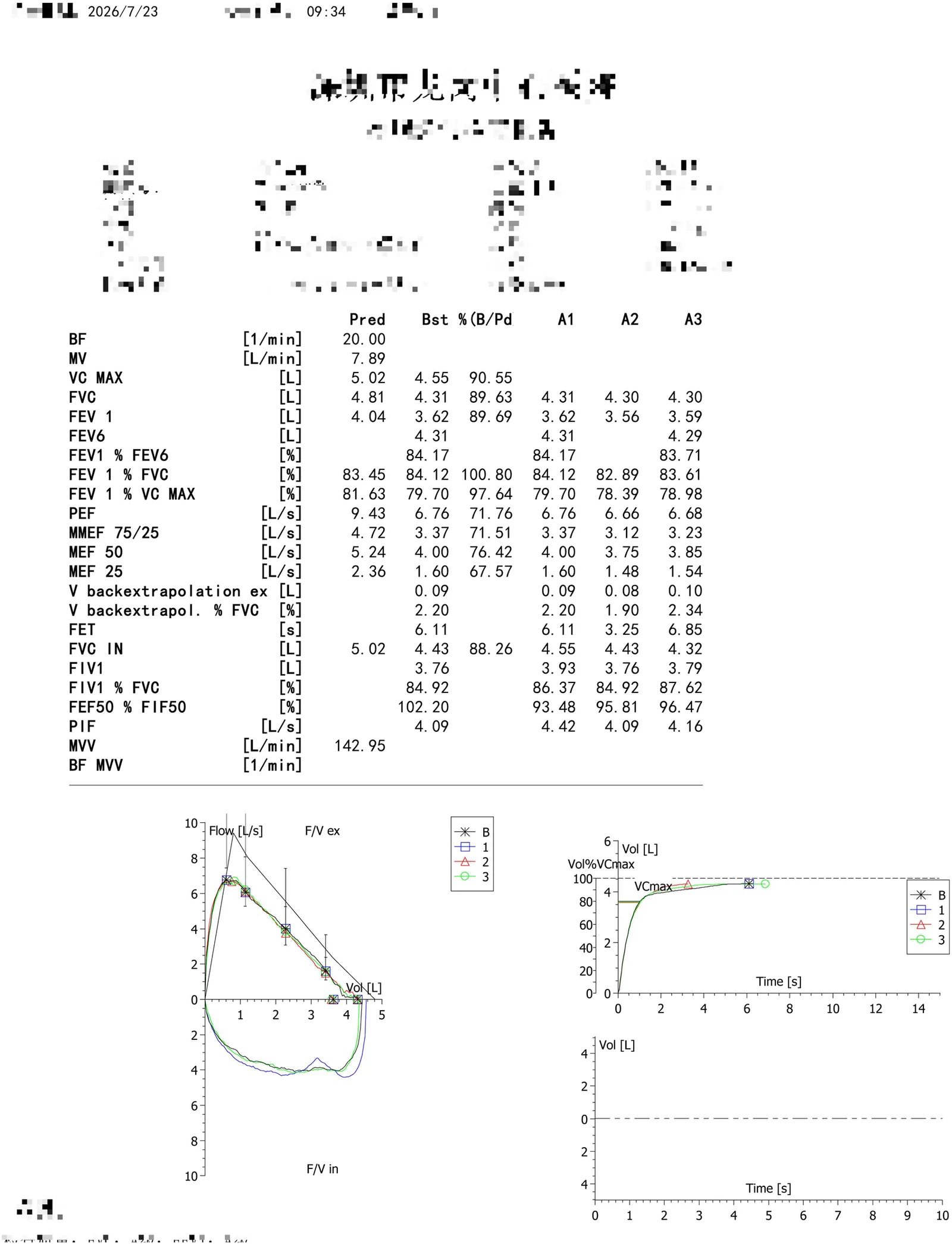
Postoperative spirometry flow-volume loop at 1-year follow-up showing complete normalization of the loop morphology. The plateau pattern observed preoperatively (Figure 1) had fully resolved.

## Patient perspective

**“**For six months, I kept coughing and felt short of breath, especially at night and when I walked fast. My doctor told me I had asthma and gave me an inhaler. I used it every day for two months, but I didn't feel any better. I was worried because I was only 30 and had never smoked. When I finally came to the hospital, the breathing test showed a strange pattern that my doctor said was not typical of asthma. They did a CT scan and found a tumor in my windpipe. I was shocked—I never expected cancer. After the surgery to remove the tumor, I could breathe normally again for the first time in months. Now, one year later, I feel completely fine and can exercise without any shortness of breath. I am grateful that my doctors did not stop at the asthma diagnosis and kept looking for the real cause.” — Patient, age 30

Timeline: see Table 1

**Table 1:** Timeline of key clinical events from symptom onset to 1-year follow-up.

Time point	Event description
Month 0	Onset of recurrent cough and progressive dyspnea
Month 1	Initial asthma diagnosis; started inhaled budesonide/formoterol (160/4.5ug, twice daily)
Month 3	No improvement after 2 months; referred to our hospital
Month 3(day 1)	Spirometry: flow-volume loop plateau pattern (fixed upper airway obstruction); bronchodilator reversibility test positive
Month 3(day 2)	Chest CT: polypoid mass at anterior wall of cervical trachea (C3-C4 level)
Month 3(day 3)	Bronchoscopy: central polypoid mass, ≈95% luminal occlusion of the cervical trachea; endoscopic electrosnare resection (diagnostic + decompressing)
Month 3(day 7)	Pathological diagnosis: LELC: NapsinA (-), TTF-1(-), Ki-67 (70%+), P40(+), CK5/6(+), Syn (-), CgA (-), EBER(ISH) (+)
Month 3(day 7–10)	Fiberoptic laryngoscopy and nasoendoscopy: no nasopharyngeal lesion;
	PET-CT: no nasopharyngeal or distant metastasis, residual mediastinal nodes
Month 4	VATS tracheal resection-anastomosis with lymphadenectomy; pathology: R0 resection (closest margin approximately 0.2 cm), 11 nodes, 2 positive (2R-1LN:1/1, 4R-5LN:1/1), invasion into cricoid cartilage, pT2N2M0 Stage IIIA
Month 5–12	Close surveillance; no adjuvant therapy
Month 12	Spirometry: complete normalization (FEV₁ 3.62L, +298%; PEF 6.76L/s, +233%); asymptomatic; no recurrence

This timeline is presented in accordance with the CARE guidelines.

## Discussion

Primary tracheal LELC is an exceptionally rare malignancy. To date, only sporadic cases have been documented in the English literature (4, 5). Among the previously published cases identified in our literature search, the youngest reported patient was a 22-year-old male. Our case of a 30-year-old male adds to the limited literature and highlights the diagnostic value of flow-volume loop plateau pattern in unmasking central airway obstruction caused by this rare tumor. Previously reported tracheal LELC cases have primarily emphasized histopathological and EBV-related features (6), with limited attention to pulmonary function findings.

### Differential diagnosis and diagnostic reasoning

The differential diagnosis of central airway obstruction in a young patient with wheezing and dyspnea includes asthma, vocal cord dysfunction/inducible laryngeal obstruction (ILO), tracheal stenosis (post-infectious or post-intubation), foreign body aspiration, and other tracheal neoplasms (e.g., squamous cell carcinoma, adenoid cystic carcinoma, carcinoid tumor, leiomyoma). In our patient, the absence of atopy, normal eosinophil count (0.08 × 10⁹/L), and lack of response to two months of inhaled corticosteroid therapy argued against primary asthma. The biphasic flow-volume loop plateau was more consistent with a fixed central airway obstruction than with isolated inducible laryngeal obstruction, which typically affects the inspiratory limb. However, flow-volume loop analysis alone cannot definitively exclude inducible laryngeal obstruction.

The biphasic flow-volume loop (a plateau in both the inspiratory and expiratory limbs) observed in this case is physiologically attributed to a fixed upper airway obstruction caused by the cervical tracheal tumor. A fixed upper airway obstruction typically presents as a flattened plateau in both the inspiratory and expiratory limbs. This characteristic morphological pattern underscores the importance of integrating flow-volume loop analysis with anatomical imaging when evaluating cervical tracheal pathologies.

Importantly, although the bronchodilator reversibility test was positive [pre-bronchodilator FEV₁ 0.91 L [22.1% predicted] vs. post-bronchodilator FEV₁ 1.75 L [42.6% predicted]; absolute increase 0.84 L, 92% increase], this did not exclude central airway obstruction. In patients with a central airway lesion, bronchodilators may improve FEV₁ through effects on peripheral airways, but the **characteristic flow-volume loop morphology—specifically the biphasic plateau—persists and is diagnostic of fixed airway obstruction**. The persistence of this pattern despite bronchodilator administration was the key observation that redirected the diagnostic workup toward imaging and bronchoscopy.

### Therapeutic strategy and rationale

The initial endoscopic resection was performed as a **diagnostic and airway-decompressing procedure** to relieve near-complete tracheal obstruction and obtain tissue for pathological diagnosis; it was not intended as definitive treatment. After the diagnosis of LELC was confirmed and PET-CT revealed residual mediastinal nodal disease, the patient underwent **curative-intent VATS tracheal resection-anastomosis with systematic lymphadenectomy**.

Adjuvant systemic therapy or radiotherapy was not administered in this patient. This was an individualized, multidisciplinary decision made in the context of an exceptionally rare tumor and the absence of disease-specific evidence. Specifically: (i) the patient achieved R0 resection with negative surgical margins; (ii) there is currently no established standard adjuvant therapy for primary tracheal LELC due to its extreme rarity; (iii) the potential long-term toxicities of adjuvant radiotherapy and chemotherapy in a young patient were considered significant; and (iv) after multidisciplinary tumor board discussion, the patient chose close surveillance. Importantly, this decision should not be interpreted as a general recommendation to omit adjuvant treatment in patients with mediastinal nodal involvement; rather, it reflects an individualized approach in the absence of high-level evidence for this ultra-rare disease.

### Immunotherapy and EBV-DNA: current evidence and limitations

Recent advances have shown that PD-1/PD-L1 inhibitors have efficacy in **locally advanced or metastatic pulmonary LELC (**6, 7); however, there is **no evidence** to support routine adjuvant immunotherapy after R0 resection of primary tracheal LELC, and this remains **strictly investigational**. Similarly, plasma EBV-DNA thresholds (e.g., >1,000 copies/mL) are **extrapolated from pulmonary LELC** (8) **and nasopharyngeal carcinoma** (9) and have **not been validated** for tracheal LELC, so they should be interpreted with caution and used only as **exploratory surveillance tools**. In our patient, the low-level detectable EBV DNA (<500 copies/mL) postoperatively prompted continued surveillance, but this finding alone did not constitute an indication for intervention.

The teaching point of this case extends beyond its rarity. In young, non-smoking patients with asthma-like symptoms, the failure to respond to an adequate trial of inhaled corticosteroids (typically 4–6 weeks) should prompt reconsideration of the initial diagnosis. The flow-volume loop: an underutilized, non-invasive, and cost-effective tool-provides a rapid means to identify patterns suggestive of central airway obstruction. A plateau pattern in both inspiratory and expiratory limbs is characteristic of fixed upper airway obstruction, which can occur at any level of the trachea, including the cervical trachea. This simple distinction can guide imaging and bronchoscopic evaluation without exposing patients to unnecessary or escalating antiasthma therapy.

This single-case study has limited generalizability. Only one-year follow-up fails to assess long-term recurrence risk. Complete molecular profiling was absent, and unified EBV-DNA monitoring criteria remain lacking.

## Conclusion

Primary tracheal LELC is a rare but treatable malignancy that can masquerade as asthma. In young, non-smoking patients with persistent or atypical asthma-like symptoms, poor response to standard inhaled therapy, audible stridor, or abnormal flow-volume loop morphology, spirometry with flow-volume loop analysis is a low-cost, high-yield diagnostic maneuver that should not be deferred.

## Data Availability

The original contributions presented in the study are included in the article/Supplementary Material, further inquiries can be directed to the corresponding author.
